# Three new species of European *Platypalpus* (Diptera, Hybotidae)

**DOI:** 10.3897/zookeys.470.8967

**Published:** 2015-01-12

**Authors:** Miroslav Barták, Štěpán Kubík

**Affiliations:** 1 Department of Zoology and Fisheries, Faculty of Agrobiology, Food and Natural Resources, Czech University of Life Sciences Prague, CZ-16521 Prague 6-Suchdol, Czech Republic

**Keywords:** *Platypalpus*, new species, Europe, key, faunistic records

## Abstract

*Platypalpus graecoides* **sp. n.** (Italy), *Platypalpus pyreneensis* **sp. n.** (Andorra), and *Platypalpus silvahumidus* **sp. n.** (Czech Republic) are described. All three species are illustrated and keyed. *Platypalpus hallensis* Grootaert & Stark, 1997 is first reported from France and Spain.

## Introduction

The genus *Platypalpus* Macquart, 1827 (Diptera: Hybotidae) belongs to the subfamily Tachydromiinae (Sinclair and Cumming 2006). It is a megadiverse genus with more than 550 species described worldwide and some 240 species known from Europe (Yang et al. 2007). The ecologically opportunistic predatory behaviour enables many species to occupy even drastically disturbed habitats, for example agricultural fields or home gardens where they often represent the commonest members of Empidoidea with supposed impact on bioregulation of pests.

## Material and method

The material studied is deposited in the collection of the Czech University of Life Sciences Prague.

Genitalia together with 2–3 pregenital segments were removed from the rest of body by means of small scissors and macerated in potassium hydroxide solution (approx. 10%) in small vials submerged into a cup of hot water for 1–2 hours. After neutralizing with 8% acetic acid the genitalia were dissected in glycerine and parts (right and left epandrial lamellae in lateral views and cerci in dorsal view) were photographed by means of an Olympus E-41 digital camera mounted on an Olympus BX51 compound microscope and images were edited with the computer software Quick Foto micro 2.3 provided with Deep focus 3.1. Each image resulted usually from combining 7–15 layers. Images were improved by means of Adobe Photoshop and they served as models for outline of hand drawn illustrations; details were added by direct observing genitalia.

The morphological terms used here follow Merz and Haenni (2000), Sinclair (2000) and Sinclair and Cumming (2006). All body measurements (including body and setae length) were taken from dry specimens (therefore the actual length may differ) by means of ocular micrometer with a Nikon SMZ 1500 binocular microscope. Male body length was measured from antennal base to the tip of the genitalia and female body length from the base of antennae to the tip of the cerci.

## Taxonomic account

### Platypalpus

Taxon classificationAnimaliaDipteraHybotidae

Macquart

Coryneta Meigen, 1800: 27. Type-species: *Musca cursitans* Fabricius, 1775, by subsequent designation of Engel (1939: 43). Suppressed by I.C.Z.N. 1963: 339 (Opinion 678).
Platypalpus Macquart, 1827: 92. Type-species: *Musca cursitans* Fabricius, 1775, by subsequent designation of Westwood (1840: 132).
Phoroxypha Rondani, 1856: 146. Type-species: *Tachydromia longicornis* Meigen, 1822, by original designation.
Cleptodromia Corti, 1907: 101 (as subgenus of *Tachydromia*). Type-species: Tachydromia (Cleptodromia) longimana Corti, 1907 by monotypy.
Brevios Brunetti, 1913: 22. Type-species: *Brevios longicornis* Brunetti, 1913 by original designation.
Howlettia Brunetti, 1913: 23. Type-species: *Howlettia flavipes* Brunetti, 1913 by monotypy.
Tachydromia , authors, not Meigen, 1803, misidentifications.
Charadrodromia Melander, 1928. Type species: *Charadrodromia microphona* Melander, 1928, by original designation.

#### Diagnosis.

Very small to medium large species with body size varying from about 1.0 to 6.0 mm, recognised by the following combination of characters: eyes separated in both sexes, without ommatrichia; postpronotal lobe well differentiated; scutum usually distinctly longer than broad; mid leg raptorial (mid femur usually thickened and armed with two rows of spine-like setae ventrally, mid tibia usually with more or less prominent ventroapical spur); wing with cell cup present.

### Taxonomy

#### Platypalpus graecoides sp. n.

Taxon classificationAnimaliaDipteraHybotidae

http://zoobank.org/47CCA475-923C-4E93-9EDA-2CB02F54790C

Figs 1–3 , 7

##### Material examined.

**Holotype:** male, Italy, Weisslahnbad, edge of forest, 1400 m, 46°28'40"N, 11°34'11"E, 4.vii.2011, M. Barták. **Paratypes:** 3 males, 7 females, same data as holotype. Holotype and paratypes are deposited in the collection of the Czech University of Life Sciences Prague.

##### Diagnosis.

The species from *Platypalpus pallidiventris* – *cursitans* group, small black with 1 pair of vertical setae, black antennae with very short postpedicel and much longer stylus; clypeus lustrous; thorax microtrichose, katepisternum broadly lustrous; legs black, mid femur strongly thickened with pale posteroventrals; long and sharply pointed apical spur on mid tibia.

##### Description.

**Male.** Head black, rather dark grey microtrichose, face more silvery. Frons ≈ 0.05 mm broad just above antennae and ≈ 0.08 mm broad in the level of fore ocellus. Face ≈ 0.03 mm broad at middle. Clypeus lustrous. Gena narrow and lustrous. Antenna black, postpedicel very short, 1.5–1.9× longer than broad, stylus 2.4–3.0× longer than postpedicel. Palpus brown, ovoid, short (about 1/2 as long as proboscis), with several long white setae. Ocellar setae black and short (≈ 0.10 mm), posterior pair half as long. A single pair of short (about as long as ocellars) black vertical setae inserted wide apart (≈ 0.16 mm). Occiput sparsely and black setose dorsally and long white setose ventrally. Proboscis half as long as head, brownish black.

Thorax black, rather dark grey microtrichose in dorsal view, pleuron lighter grey microtrichose, katepisternum with large lustrous patch leaving only narrow posterior margin microtrichose. Large thoracic setae including acrostichals and dorsocentrals black or at least very dark brown, hairs on proepisternum, legs, squama and wing mostly white. Chaetotaxy: postpronotal seta rather short but strong; acrostichals biserial and short (≈ 0.08 mm in middle of rows), about 6–10 setae in one row; dorsocentrals similarly short and uniserial, 6–10 setae in one row, last pair strong and long; notopleuron with 2 long black setae on posterior part (upper seta usually longer than lower one) and also with several additional much smaller setae; 1 postalar and 1 pair of apical scutellar setae in addition to another much smaller lateral pair.

Wing clear with yellowish brown veins. Veins R_4+5_ and M_1_ nearly straight and almost parallel. Crossveins separated. Vein Cu almost straight, anal vein depigmented but visible along its length. Vein R_1_ forming large swollen area along confluence with costa. A single strong black costal seta. Squama yellow with pale fringes. Halter pale yellow.

Legs black, only knees of fore leg and base of fore tarsus (and sometimes also bases of mid and hind tarsi) paler, dark yellowish brown. Tarsi without apparent annulations. Fore femur slightly thickened, with two ventral rows of white setae, those in posteroventral row up to as long as depth of femur. Fore tibia very slightly thickened, short setose. Mid femur thickened (≈ 0.25 mm broad at broadest point), with rather short pale posteroventrals. Mid tibia with very long (twice longer than width of tibia) sharply pointed apical spur. Hind leg slender, without conspicuous setation.

**Figures 1–3.**
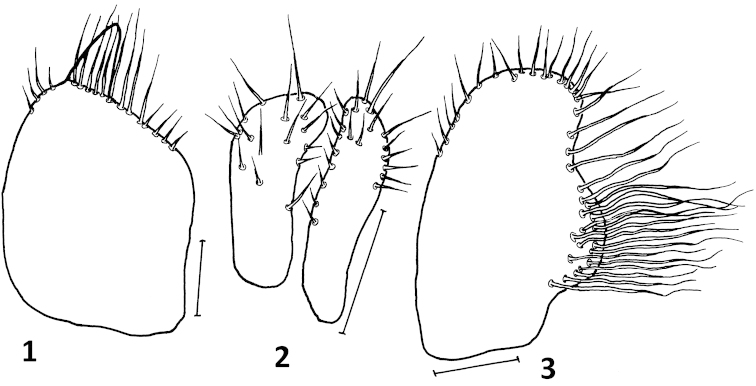
*Platypalpus graecoides* sp. n.: **1** right epandrial lamella **2** cerci **3** left epandrial lamella. Scales = 0.10 mm.

Abdomen blackish brown, lustrous, including dorsum of tergite 1, sides of basal tergites with microtrichose stripes: on tergite 1 complete, on tergite 2 half width of tergite, on remaining tergites much narrower; sparsely covered with short pale setae somewhat longer on last 2–3 tergites. Genitalia (Figs 1–3) with left epandrial lamella armed with about 30 long wavy setae on small protuberance, right epandrial lamella with several setae apically, cerci small, simple and concealed within lamellae.

**Female.** Legs slightly paler than in male, especially basal parts of tarsi almost brownish yellow. Abdomen lustrous, microtrichosity on basal tergites as in male, last two segments entirely microtrichose

**Length.** body 1.8–2.4 mm, wing 2.0–2.6 mm.

##### Etymology.

The specific epitheton stresses similarity with *Platypalpus graecus* Grootaert & Chvála, 1992.

##### Distribution.

Italy.

##### Remarks.

The species described above is similar to *Platypalpus graecus* and it may be identified according to the key below. Differences are as follows: *Platypalpus graecus* is somewhat larger (wing 2.6–3.0 mm), with microtrichose clypeus, longer postpedicel and shorter arista, with a polished line between mesonotum and postpronotum, with long hairs on metatarsi, with short anterior black bristles on mid femur, without costal seta, with abdomen more lustrous (only indistinct lateral patch on first tergite), and slightly different genitalia (apical setae on right epandrial lamella arranged in two separate tufts). Moreover, *Platypalpus graecus* was taken at Pyrgos (Greece) lying in lowland near seashore but *Platypalpus graecoides* sp. n. originates from high Dolomites mountains.

#### Platypalpus pyreneensis sp. n.

Taxon classificationAnimaliaDipteraHybotidae

http://zoobank.org/8A110E82-8F4A-4B27-8F09-495CA04A5D4C

Figs 4–6 , 8

##### Material examined.

**Holotype:** male, Andorra, Pto. de Envalira, subalpine meadow, 2200 m, 42°32'N, 1°43"E, 8.vii.1990, M. Barták. **Paratypes:** 3 males, 3 females, same data as holotype; 1 male, same locality, pine wood nr. brook, 1800 m, 42°33'N, 1°42'E, M. Barták. Holotype and paratypes are deposited in the collection of the Czech University of Life Sciences Prague.

##### Diagnosis.

The species from *Platypalpus pallidiventris* – *cursitans* group, small black, with 1 pair of vertical setae, black antennae with short postpedicel and much longer stylus; thorax microtrichose, dorsocentrals long and bristle like; katepisternum broadly lustrous; legs black, mid femur strongly thickened with pale posteroventrals; long and sharply pointed apical spur on mid tibia.

##### Description.

**Male.** Head black, rather light grey microtrichose. Frons rather thinly microtrichose, ≈ 0.06 mm broad just above antennae and ≈ 0.11 mm broad in the level of fore ocellus. Face ≈ 0.04 mm broad at middle. Clypeus microtrichose. Gena narrow and lustrous. Antenna black, postpedicel short, 1.6–2.3× longer than broad, stylus 2.3–3.1× longer than postpedicel. Palpus brown, ovoid, and short (less than 1/2 as long as proboscis), with several long white setae. Ocellar setae black and rather long (≈ 0.15 mm), posterior pair half as long. A single pair of long (≈ 0.17 mm) black vertical setae inserted wide apart (≈ 0.18 mm). Occiput sparsely and black setose dorsally and long white setose ventrally. Proboscis slightly more than half as long as head, brownish black.

Thorax black, rather thinly grey microtrichose in dorsal view, pleuron lighter grey microtrichose, katepisternum with large lustrous patch leaving only narrow hind margin microtrichose. Large thoracic setae including bristle like dorsocentrals black or at least very dark brown, hairs including smaller acrostichals paler, brown to whitish yellow. Chaetotaxy: postpronotal seta yellowish to black, long; acrostichals biserial and short (≈ 0.08 mm in middle of rows), about 5–8 setae in one row; dorsocentrals uniserial, strong, varying in length from ≈ 0.12 mm to ≈ 0.20 mm, 5–7 setae in one row, sometimes with additional paler and smaller hairs between them, last pair somewhat longer; notopleuron with 2–3 long setae (anterior seta present or absent) and with several additional much smaller and paler seta(e); 1 postalar and 1 pair of scutellar setae in addition to another much smaller and paler pair.

Wing very slightly brownish with brown veins. Veins R_4+5_ and M_1_ nearly straight and almost parallel. Crossveins narrowly separated. Vein Cu almost straight, anal vein depigmented but visible along its length. A single long black costal seta. Squama yellow with pale fringes. Halter pale yellow.

Legs black, only knees of fore leg yellowish brown. Tarsi somewhat paler in some specimens, without apparent annulations. Fore femur slightly thickened, with two ventral rows of white setae, those in posteroventral row up to as long as depth of femur. Fore tibia very slightly thickened, with two dorsal black setae and elongated ventral white hairs longer than depth of tibia. Mid femur thickened (≈ 0.22 mm broad at broadest point), with rather long white anteroventrals and usually with 2 strong black anterior setae, posteroventrals long and white. Mid tibia with long sharply pointed apical spur. Hind legs slender, without conspicuous setation, hind tibia in some specimens with several dorsal setae.

**Figures 4–6.**
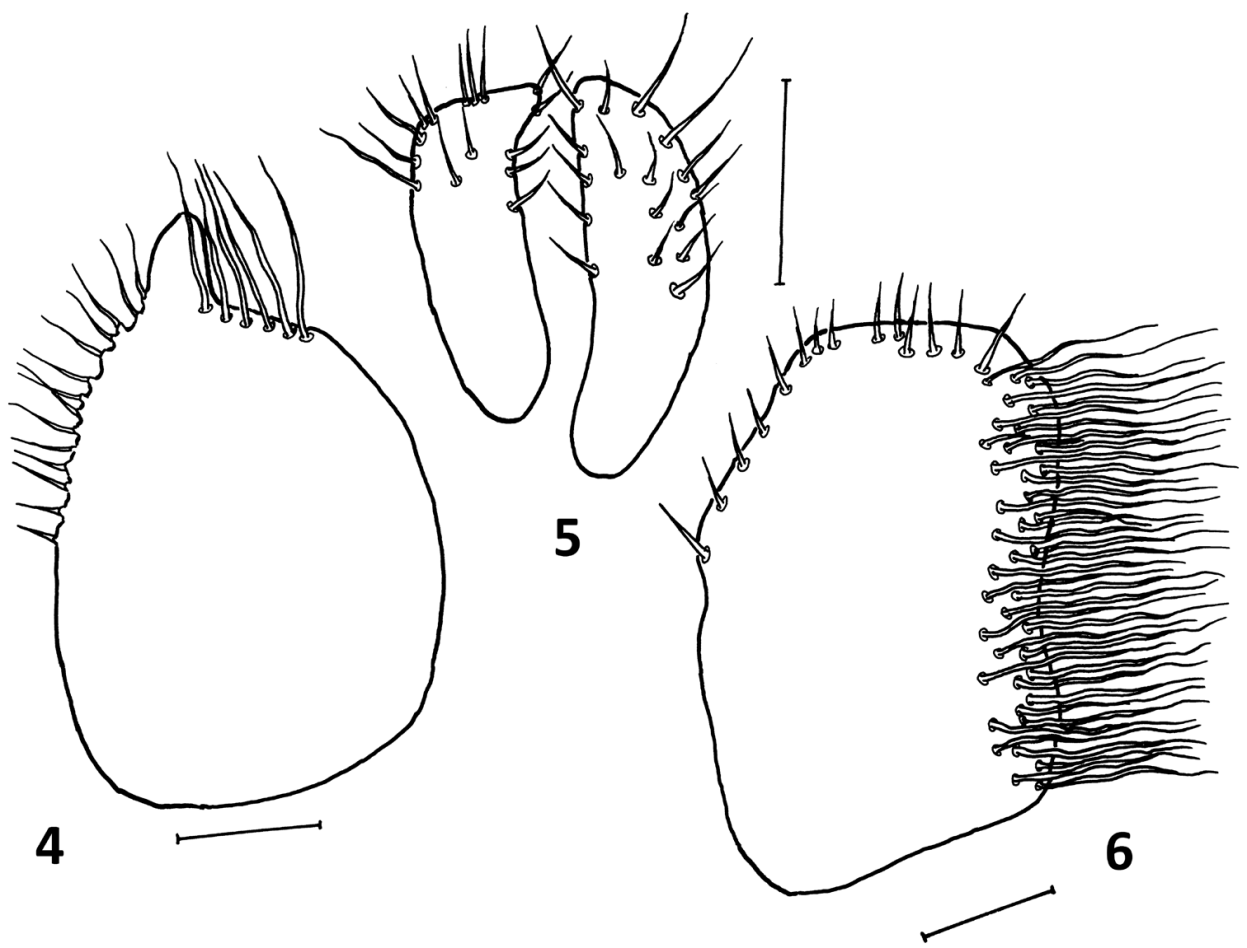
*Platypalpus pyreneensis* sp. n.: **4** right epandrial lamella **5** cerci **6** left epandrial lamella. Scales = 0.10 mm.

Abdomen blackish brown, lustrous, including dorsum of tergite 1, sides of first tergite entirely microtrichose, second tergite with narrow microtrichose stripe; sparsely covered with short pale setae somewhat longer on last 2–3 segments. Genitalia (Figs 4–6) with left epandrial lamella bearing some 50 long setae distributed along whole ventral margin, right epandrial lamella with strong setae both apically and ventrally and cerci small and simple, concealed within lamellae.

**Figures 7–9.**
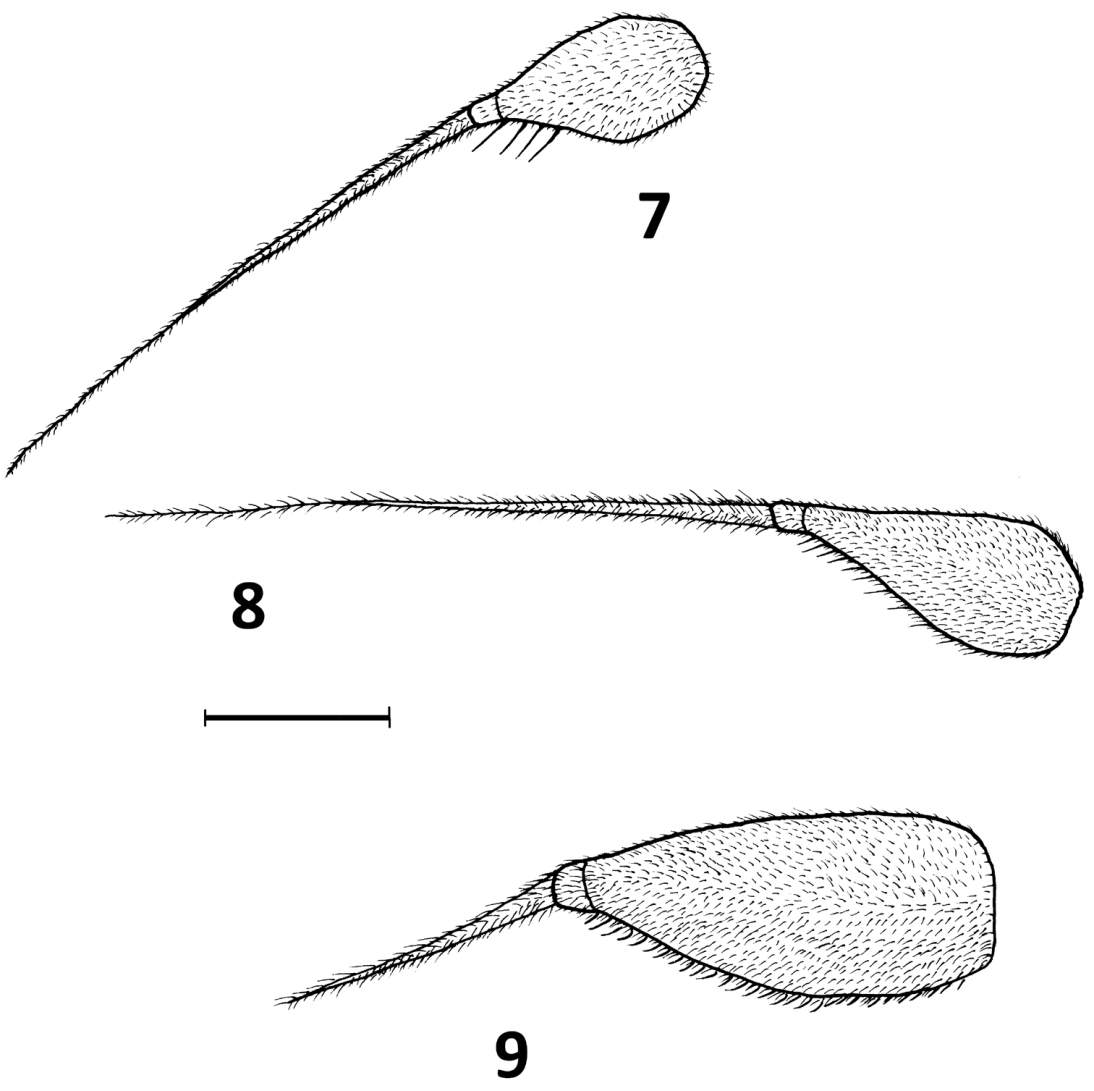
Antennae: **7** *Platypalpus graecoides* sp. n., **8** *Platypalpus pyreneensis* sp. n. **9** *Platypalpus silvahumidus* sp. n. Scale = 0.10 mm.

**Female.** Abdomen lustrous, microtrichosity on basal two tergites as in male, last two segments entirely microtrichose

**Length.** body 2.4–2.6 mm, wing 2.4–2.6 mm.

##### Etymology.

The specific epitheton is derived from mountain range name (Pyrenees).

##### Distribution.

Andorra.

##### Remarks.

The species described above may be arranged with difficulties in the key by Grootaert and Chvála (1992) because of confusion in section 99: several specimens of the newly described species have only two notopleural setae but several have three black and strong setae so this character must be considered variable. So we propose to modify section 99 of the key as follows:

**Table:** 

99 (94)	Coxae, femora and tibiae entirely black, only fore knees may be paler. Abdominal sternites lustrous. Postpedicel at most 2.5× longer than broad	**99a**
–	Some parts of coxae, femora and tibiae at least brownish yellow, in difficult cases either: abdominal sternites microtrichose or postpedicel more than 3× longer than broad	**99c**
99a (99)	Dorsocentral setae partly long and bristle like	***Platypalpus pyreneensis* sp. n.**
–	Dorsocentral setae uniformly short and hair like	**99b**
99b (99a)	Clypeus microtrichose. Polished line between postpronotum and mesonotum. Very long posteroventral hairs on metatarsi	***Platypalpus graecus***
–	Clypeus lustrous. No polished line between postpronotum and mesonotum. Short posteroventral hairs on metatarsi	***Platypalpus graecoides* sp. n.**
99c (99)	Three bristles on notopleuron	**100**
–	Two bristles on notopleuron	**103 (except *Platypalpus graecus* )**

#### Platypalpus silvahumidus sp. n.

Taxon classificationAnimaliaDipteraHybotidae

http://zoobank.org/A69DFC44-53FD-458F-94CA-773271DB204F

Fig. 9

##### Material examined.

**Holotype:** female, Czech Republic, Podyjí, Devět mlýnů, floodplain wood, MT (= Malaise trap), 270 m, 48°49'07"N, 15°58'17"E, 29.iv.-21.v.2004, M. Barták & Š. Kubík. **Paratypes:** 2 females, same data as holotype; 1 female, same locality as holotype, 21.v.–12.vi.2004; 2 females, Podyjí NP, pod Šobesem, wetland nr. river, MT, 48°48'48"N, 15°58'51"E, 26.iii.–8.v.2002, M. Barták & Š. Kubík; 2 females, same locality, 8.–22.v.2002; 1 female, Podyjí NP, Faltýskův mlýn, wetland nr. river, MT, 48°50'43"N, 15°54'08"E, 29.iv.–21.v.2004, M. Barták & Š. Kubík; 1 female, Vykáň, floodplain wood, sweeping, 190 m, 50°07'00"N, 14°48'33"E, 1.v.2007, M. Barták. Holotype and paratypes are deposited in the collection of the Czech University of Life Sciences Prague.

##### Diagnosis.

Black species of *Platypalpus hackmani* group with 2 pairs of vertical setae, black antennae with broad postpedicel; thorax microtrichose including katepisternum, most setae black; legs dark to brownish yellow, mid femur narrower than fore femur, with only slightly developed ventral setae, mid tibia without apical spur; abdomen lustrous.

##### Description.

**Female.** Head black, brownish grey microtrichose. Frons ≈ 0.09 mm broad just above antennae and ≈ 0.13 mm broad in the level of fore ocellus. Face ≈ 0.06 mm broad at middle. Clypeus microtrichose. Gena microtrichose and broad. Antenna black, postpedicel broad (≈ 0.10 mm), 2.2–2.6× longer than broad and 1.1–1.7× longer than stylus. Palpus brown, narrow and short (about 1/3 as long as proboscis), with long black subterminal seta. Both pairs of ocellar setae black and long, anterior one ≈ 0.15 mm long, posterior one only slightly shorter. Two pairs of black vertical setae, inner very long (≈ 0.25 mm) and strong. Occiput sparsely and black setose including lower part. Proboscis 2/3 as long as head, brownish black.

Thorax brownish black, entirely brownish grey microtrichose including katepisternum. All setae black. Chaetotaxy: postpronotal seta long and strong (several additional much smaller setae present), posthumeral seta similarly long and strong (not anterior notopleural but true posthumeral seta); acrostichals biserial, slightly divergent (more on posterior part), moderately long (≈ 0.10 mm in middle of rows), about 6–9 setae in one row; dorsocentrals slightly longer (≈ 0.15 mm in middle of row), uniserial, 7–10 setae in one row, last 1–2 pairs strong and long (several acrostichals and/or dorsocentrals in some specimens distinctly longer and more bristle-like); notopleuron with 2 long setae on posterior part and an additional smaller seta; 1 long postalar and 2 pairs of scutellar setae.

Wing clear with brownish yellow veins. Veins R_4+5_ and M_1_ nearly straight and almost parallel. Crossveins contiguous. Vein Cu slightly bowed but not S-shaped, proximal part of anal vein almost invisible. Two (sometimes 3) unusually long and strong black costal setae. Squama yellow with pale fringes. Halter pale yellow.

Legs black setose, rather dark yellow including fore coxa, mid femur, mid tibia and tarsi of fore and mid legs; mid and hind coxae, hind femur apically, hind tibia and hind tarsus brownish yellow. Fore femur strongly thickened (≈ 0.25 mm at broadest point), with two rows of setae ventrally up to half as long as depth of femur. Fore tibia distinctly thickened, with 2–3 black bristly setae dorsally. Mid femur narrower than fore femur, ventral rows consisting of relatively fine and short hairs only slightly stronger than remaining setation. Mid tibia without apical spur. Hind femur short setose. Hind tibia thin, with 2 antero- and 2 posterodorsal black and strong setae slightly longer than depth of tibia.

Abdomen blackish brown, lustrous, including dorsum of tergite 1, sides of tergites 7–8 and mostly also sternite 7; microtrichose parts: sides of tergite 1, dorsum of tergites 7 and 8, sternite 8, also tergites 2–3 with narrow anterior microtrichose stripes; abdomen sparsely covered with short dark setae.

**Male.** Unknown.

**Length.** body 2.5–2.7 mm, wing 2.4–2.9 mm.

##### Etymology.

The specific epitheton originated from two Latin words depicting typical biotope of this species (silva = forest, humidus = damp) and should be treated as adjective (i.e. silvahumida, silvahumidum).

##### Distribution.

Czech Republic.

##### Remarks.

The species described above belongs to the *Platypalpus hackmani* group. In spite we have no male at our disposal, we don’t hesitate to describe it because this species is very different from all other species by characters which are probably not sexually different (it may be even parthenogenetic). According to Shamshev and Grootaert (2012) and Grootaert and Stark (1997), in the Palaearctic the *Platypalpus hackmani* group includes six species that share distinct humeri, a posthumeral bristle, usually dusted katepisternum, a more or less dusted abdomen, with a very thickened fore femur, while the mid femur is slender with only fine bristles ventrally instead of the usual double row of spine-like bristles and lacking a row of posteroventral bristles, and lacking the apical spur on the mid tibia. Apparently not all these characters are conclusive to distinguish this species group. It is difficult to place the species into the comprehensive key by Grootaert and Chvála (1992). The whole section of the key starting from number 195 is confusing due (in part) to description of two species of *Platypalpus hackmani* group with long antennae (*Platypalpus canariensis* Grootaert & Chvála, 1992 and *Platypalpus hallensis* Grootert & Stark, 1997, see also note below the latter species). We propose to adapt this section of the key as follows:

**Table:** 

195 (194)	Posthumeral seta present. Katepisternum microtrichose or with only small lustrous patch at middle. Mid femur narrower than fore femur and/or without ventral spines	**195a**
–	Posthumeral seta absent. Katepisternum with large or small lustrous patch. Mid femur broader than fore femur, bearing two rows of spine like setae ventrally	**198**
195a (195)	Large bristles on head and thorax dark, brown to black	**195b**
–	Large bristles on head and thorax pale, white to yellow	**197**
195b (195a)	Abdomen largely lustrous.	***Platypalpus silvahumidus* sp. n.**
–	Abdomen entirely microtrichose	**196**
196 (195b)	Postpedicel at least 4× longer than broad. Katepisternum with lustrous patch in middle	***Platypalpus canariensis* Grootaert & Chvála**
–	Postpedicel at most X× longer than broad. Katepisternum entirely microtrichose	***Platypalpus minutissimus* (Strobl)**
197 (195a)	Tarsi entirely yellow. Genital lamellae short setose (Chvála 1989, Figs 69–71)	***Platypalpus nanus* (Oldenberg)**
–	Last tarsal segment brown to black. Genital lamellae long setose (Grootaert and Stark 1997, Figs 3, 6, 7)	***Platypalpus hallensis* Grootaert & Stark**
198 (195)	Postpedicel at most 2× longer than broad	**199**
–	Postpedicel at least 2.5× longer than broad	**200**

### Faunistic records

*Platypalpus hallensis* Grootaert & Stark, 1997. 1 male, Spain, 5 km E of Tordesillas, on flowers in pine wood, 686 m, 41°31'02"N, 4°56'25"W, 22.v.2008, M. Barták; 1 male, France, 9 km S of Sété, seashore dunes, PT (= pan traps), 43°21'9"N, 3°45'48"E, 21.–23.v.2006, M. Barták. Both specimens very slightly differ from original description and from Central European specimens in having shorter antennae (postpedicel being twice or slightly more than twice as long as broad) and slightly shorter setose genital lamellae. However, otherwise they are identical in all details with Central European specimens and with original description. First records for both France and Spain.

## Supplementary Material

XML Treatment for Platypalpus

XML Treatment for Platypalpus graecoides

XML Treatment for Platypalpus pyreneensis

XML Treatment for Platypalpus silvahumidus
